# Space-efficient and exact de Bruijn graph representation based on a Bloom filter

**DOI:** 10.1186/1748-7188-8-22

**Published:** 2013-09-16

**Authors:** Rayan Chikhi, Guillaume Rizk

**Affiliations:** 1 Computer Science department, ENS Cachan / IRISA / INRIA, Rennes 35042, France; 2 Algorizk, Paris 75013, France

**Keywords:** de novo assembly, de Bruijn graph, Bloom filter

## Abstract

**Background:**

The de Bruijn graph data structure is widely used in next-generation sequencing (NGS). Many programs, e.g. *de novo* assemblers, rely on in-memory representation of this graph. However, current techniques for representing the de Bruijn graph of a human genome require a large amount of memory (≥30 GB).

**Results:**

We propose a new encoding of the de Bruijn graph, which occupies an order of magnitude less space than current representations. The encoding is based on a Bloom filter, with an additional structure to remove critical false positives.

**Conclusions:**

An assembly software implementing this structure, Minia, performed a complete *de novo* assembly of human genome short reads using 5.7 GB of memory in 23 hours.

## Background

The de Bruijn graph of a set of DNA or RNA sequences is a data structure which plays an increasingly important role in next-generation sequencing applications. It was first introduced to perform *de novo* assembly of DNA sequences [1]. It has recently been used in a wider set of applications: *de novo* mRNA [2] and metagenome [3] assembly, genomic variants detection [4,5] and *de novo* alternative splicing calling [6]. However, an important practical issue of this structure is its high memory footprint for large organisms. For instance, the straightforward encoding of the de Bruijn graph for the human genome (*n*≈2.4·10^9^,*k*-mer size *k* = 27) requires 15 GB (*n*·*k*/4 bytes) of memory to store the nodes sequences alone. Graphs for much larger genomes and metagenomes cannot be constructed on a typical lab cluster, because of the prohibitive memory usage.

Recent research on de Bruijn graphs has been targeted on designing more lightweight data structures. Li *et al.* pioneered minimum-information de Bruijn graphs, by not recording read locations and paired-end information [7]. Simpson *et al.* implemented a distributed de Bruijn graph to reduce the memory usage per node [8]. Conway and Bromage applied sparse bit array structures to store an implicit, immutable graph representation [9]. Targeted methods compute local assemblies around sequences of interest, using negligible memory, with greedy extensions [10] or portions of the de Bruijn graph [11]. Ye *et al.* recently showed that a graph roughly equivalent to the de Bruijn graph can be obtained by storing only one out of *g* nodes (10 ≤ *g* ≤ 25) [12].

Conway and Bromage observed that the self-information of the edges is a lower bound for exactly encoding the de Bruijn graph [9]:

log2(4k+1|E|)bits,

where *k*+1 is the length of the sequence that uniquely defines an edge, and |*E*| is the number of edges. In this article, we will consider for simplicity that a de Bruijn graph is fully defined by its nodes. A similar lower bound can then be derived from the self-information of the nodes. For a human genome graph, the self-information of |*N*|≈2.4·10^9^ nodes is log2(4k|N|)≈6.8GB for *k* = 27, i.e. ≈24 bits per node.

A recent article [13] from Pell *et al.* introduced the *probabilistic de Bruijn graph*, which is a de Bruijn graph stored as a Bloom filter (described in the next section). It is shown that the graph can be encoded with as little as 4 bits per node. An important drawback of this representation is that the Bloom filter introduces false nodes and false branching. However, they observe that the global structure of the graph is approximately preserved, up to a certain false positive rate. Pell *et al.* did not perform assembly directly by traversing the probabilistic graph. Instead, they use the graph to partition the set of reads into smaller sets, which are then assembled in turns using a classical assembler. In the arXiv version of [13] (Dec 2011), it is unclear how much memory is required by the partitioning algorithm.

In this article, we focus on encoding an exact representation of the de Bruijn graph that efficiently implements the following operations: 

1. For any node, enumerate its neighbors

2. Sequentially enumerate all the nodes

The first operation requires random access, hence is supported by a structure stored in memory. Specifically, we show in this article that a probabilistic de Bruijn graph can be used to perform the first operation exactly, by recording a set of troublesome false positives. The second operation can be done with sequential access to the list of nodes stored on disk. One highlight of our scheme is that the resulting memory usage is approximated by

$$1.44\log_{2}(\frac{16k}{2.08})+2.08\;\text{bits}/k\text{-mer}.$$

For the human genome example above and *k* = 27, the size of the structure is 3.7 GB, i.e. 13.2 bits per node. This is effectively below the self-information of the nodes. While this may appear surprising, this structure does not store the precise set of nodes in memory. In fact, compared to a classical de Bruijn graph, the membership of an arbitrary node cannot be efficiently answered by this representation. However, for the purpose of many applications (e.g. assembly), these membership queries are not needed.

We apply this representation to perform *de novo* assembly by traversing the graph. In our context, we refer by traversal to any algorithm which visits all the nodes of the graph exactly once (e.g. a depth-first search algorithm). Thus, a mechanism is needed to mark which nodes have already been visited. However, nodes of a probabilistic de Bruijn graph cannot store additional information. We show that recording only the visited complex nodes (those with in-degree or out-degree different than one) is a space-efficient solution. The combination of (i) the probabilistic de Bruijn graph along with the set of critical false positives, and (ii) the marking scheme, enables to perform very low-memory *de novo* assembly.

In the first Section, the notions of de Bruijn graphs and Bloom filters are formally defined. Section “Removing critical false positives” describes our scheme for exactly encoding the de Bruijn graph using a Bloom filter. Section “Additional marking structure for graph traversal” presents a solution for traversing our representation of the de Bruijn graph. Section “Results and discussion” presents two experimental results: (i) an evaluation of the usefulness of removing false positives and (ii) an assembly of a real human dataset using an implementation of the structure. A comparison is made with other recent assemblers based on de Bruijn graphs.

## de Bruijn graphs and Bloom filters

The **de Bruijn graph**[1], for a set of strings *S*, is a directed graph. For simplicity, we adopt a node-centric definition. The nodes are all the *k*-length substrings (also called *k-mers*) of each string in *S*. An edge *s*_1_→*s*_2_ is present if the (*k*−1)-length suffix of *s*_1_ is also a prefix of *s*_2_. Throughout this article, we will indifferently refer to a node and its *k*-mer sequence as the same object.

A more popular, edge-centric definition of de Bruijn graphs requires that edges reflect consecutive nodes. For *k* ′-mer nodes, an edge *s*_1_→*s*_2_ is present if there exists a (*k*^′^+1)-mer in a string of *S* containing *s*_1_ as a prefix and *s*_2_ as a suffix. The node-centric and edge-centric definitions are essentially equivalent when *k*^′^=*k*−1 (although in the former, nodes have length *k*, and *k*−1 in the latter).

The **Bloom filter**[14] is a space efficient hash-based data structure, designed to test whether an element is in a set. It consists of a bit array of *m* bits, initialized with zeros, and *h* hash functions. To insert or test the membership of an element, *h* hash values are computed, yielding *h* array positions. The insert operation corresponds to setting all these positions to 1. The membership operation returns *yes* if and only if all of the bits at these positions are 1. A *no* answer means the element is definitely not in the set. A *yes* answer indicates that the element may or may not be in the set. Hence, the Bloom filter has one-sided errors. The probability of false positives increases with the number of elements inserted in the Bloom filter. When considering hash functions that yield equally likely positions in the bit array, and for large enough array size *m* and number of inserted elements *n*, the false positive rate F is [14]:

(1) $$\begin{array}{l} F\;\approx \;{\left(1-e^{-\text{hn}/m}\right)}^{h}={\left(1-e^{-h/r}\right)}^{h} \end{array}$$

where *r* = *m*/*n* is the number of bits per element. For a fixed ratio *r*, minimizing Equation 1 yields the optimal number of hash functions *h*≈0.7*r*, for which F is approximately 0.6185^*r*^. Solving Equation 1 for *m*, assuming that *h* is the optimal number of hash function, yields $m\approx 1.44\log_{2}(\frac{1}{F})n$.

The **probabilistic de Bruijn graph** is obtained by inserting all the nodes of a de Bruijn graph (i.e all *k*-mers) in a Bloom filter [13]. Edges are implicitly deduced by querying the Bloom filter for the membership of all possible extensions of a *k*-mer. Specifically, an *extension* of a *k*-mer *v* is the concatenation of either (i) the *k*−1 suffix of *v* with one of the four possible nucleotides, or (ii) one of the four nucleotides with the *k*−1 prefix of *v*.

The probabilistic de Bruijn graph holds an over-approximation of the original de Bruijn graph. Querying the Bloom filter for the existence of an arbitrary node may return a false positive answer (but never a false negative). This introduces false branching between original and false positive nodes.

## Removing critical false positives

### The *cFP* structure

Our contribution is a mechanism that avoids false branching. Specifically, we propose to detect and store false positive elements which are responsible for false branching, in a separate structure. To this end, we introduce the *cFP* structure of *critical False Positivesk*-mers, implemented with a standard set allowing fast membership test. Each query to the Bloom filter is modified such that the *yes* answer is returned if and only if the Bloom filter answers *yes* and the element is not in *cFP*.

Naturally, if *cFP* contained all the false positives elements, the benefits of using a Bloom filter for memory efficiency would be lost. The key observation is that the *k*-mers which will be queried when traversing the graph are not *all* possible *k*-mers. Let S be the set of true positive nodes, and E be the set of extensions of nodes from S. Assuming we only traverse the graph by starting from a node in S, false positives that do not belong to E will never be queried. Therefore, the set *cFP* will be a subset of E. Let P be the set of all elements of E for which the Bloom filter answers *yes*. The **set of critical false positives*cFP*** is then formally defined as *cFP*=P∖S.

**Figure 1:**
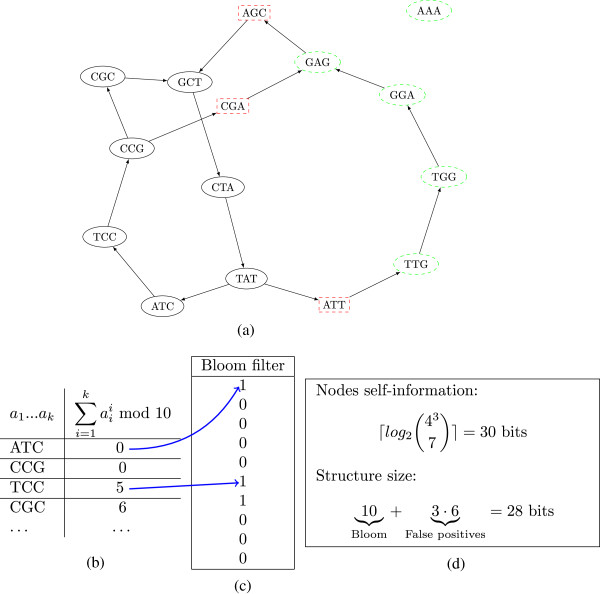
**A complete example of removing false positives in the probabilistic de Bruijn graph. (a)** shows S, an example de Bruijn graph (the 7 non-dashed nodes), and ℬ, its probabilistic representation from a Bloom filter (taking the union of all nodes). Dashed rectangular nodes (in red in the electronic version) are immediate neighbors of S in ℬ. These nodes are the critical false positives. Dashed circular nodes (in green) are all the other nodes of ℬ; **(b)** shows a sample of the hash values associates to the nodes of S (a toy hash function is used); **(c)** shows the complete Bloom filter associated to S; incidentally, the nodes of ℬ are exactly those to which the Bloom filter answers positively; **(d)** describes the lower bound for exactly encoding the nodes of S (self-information) and the space required to encode our structure (Bloom filter, 10 bits, and 3 critical false positives, 6 bits per 3-mer).

Figure 1 shows a simple graph with the set S of correct nodes in regular circles and *cFP* in dashed rectangles. The exact representation of the graph is therefore made of two data structures: the Bloom filter, and the set *cFP* of critical false positives. Algorithm 1 describes how to construct *cFP* using a fixed amount of memory. The set P is created on disk, from which *cFP* is then gradually constructed by iteratively filtering P with partitions of S ((*D*_*i*_)_*i*≥0_) loaded in a hash-table. The sets S, P, and (*D*_*i*_)_*i*≥0_ are stored on the hard disk. The sets (*P*_*i*_)_*i*≥0_ reside in RAM, and are dimensioned to occupy as much space as the Bloom filter (which was freed at Step 4). Note that I/O to the disk are always sequential.

Algorithm 1 Constant-memory enumeration of critical false positives

### Dimensioning the Bloom filter for minimal memory usage

The set *cFP* grows with the number of false positives. To optimize memory usage, a trade-off between the sizes of the Bloom filter and *cFP* is studied here.

Using the same notations as in the definition of the Bloom filter, given that n=|S|, the size of the filter *m* and the false positive rate F are related through Equation 1. The expected size of *cFP* is 8n·F, since each node only has eight possible extensions, which might be false positives. In the encoding of *cFP*, each *k*-mer occupies 2·*k* bits. Recall that for a given false positive rate F, the expected optimal Bloom filter size is $1.44n\log_{2}(\frac{1}{F})$. The total structure size is thus expected to be

(2) $$\begin{array}{l} \underset{\text{Bloom filter}}{\underset{︸}{1.44n\log_{2}\left(\frac{1}{F}\right)}}+\underset{\text{cFP}}{\underset{︸}{\left(16\cdot \mathrm{Fnk}\right)}}\;n\;\text{bits} \end{array}$$

The size is minimal for $F\approx {\left(16k/2.08\right)}^{-1}$. Thus, the minimal number of bits required to store the Bloom filter and the set *cFP* is approximately

(3) $$\begin{array}{lcrr} n\cdot (1.44\log_{2}(\frac{16k}{2.08})+2.08). & & & \end{array}$$

**Figure 2:**
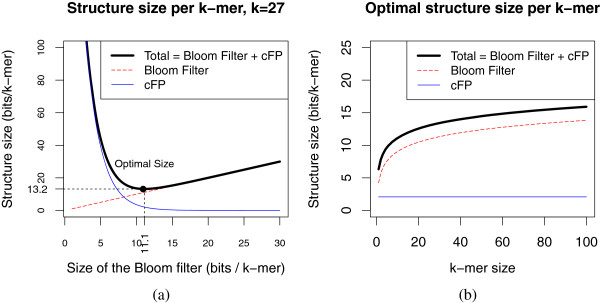
**Optimal data structure size for the parameter*r*, then for the parameter*k*.(a)** Structure size (Bloom filter, critical false positives) in function of the number of bits per *k*-mer allocated to the Bloom filter (also called ratio *r*) for *k* = 27. The trade-off that optimizes the total size is shown in dashed lines. **(b)** Optimal size of the structure for different values of *k*.

For illustration, Figure 2-(a) shows the size of the structure for various Bloom filter sizes and *k* = 27. For this value of *k*, the optimal size of the Bloom filter is 11.1 bits per *k*-mer, and the total structure occupies 13.2 bits per *k*-mer. Figure 2-(b) shows that *k* has only a modest influence on the optimal structure size. Note that the size of the *cFP* structure is in fact independent of *k*.

In comparison, a Bloom filter with virtually no critical false positives would require F·8n<1, i.e. *r* > 1.44 log2(8*n*). For a human genome (*n* = 2.4·10^9^), *r* would be greater than 49.2, yielding a Bloom filter of size 13.7 GB.

## Additional marking structure for graph traversal

Many NGS applications, e.g. *de novo* assembly of genomes [15] and transcriptomes [2], and *de novo* variant detection [6], rely on (i) simplifying and (ii) traversing the de Bruijn graph. However, the graph as represented in the previous section neither supports (i) simplifications (as it is immutable) nor (ii) traversals (as the Bloom filter cannot store an additional visited bit per node). To address the former issue, we argue that the simplification step can be avoided by designing a slightly more complex traversal procedure [16].

We introduce a novel, lightweight mechanism to record which portions of the graph have already been visited. The idea behind this mechanism is that not every node needs to be marked. Specifically, nodes that are inside simple paths (i.e nodes having an in-degree of 1 and an out-degree of 1) will either be all marked or all unmarked. We will refer to nodes having their in-degree or out-degree different to 1 as *complex* nodes. We propose to store marking information of complex nodes, by explicitly storing complex nodes in a separate hash table. In de Bruijn graphs of genomes, the complete set of nodes dwarfs the set of complex nodes, however the ratio depends on the genome complexity [17]. The memory usage of the marking structure is *n*_c_*C*, where *n*_c_ is the number of complex nodes in the graph and *C* is the memory usage of each entry in the hash table (*C*≈2*k*+8).

## Implementation

The de Bruijn graph structure described in this article was implemented in a new *de novo* assembly software: Minia^a^. An important preliminary step is to retrieve the list of distinct *k*-mers that appear in the reads, i.e. true graph nodes. To discard likely sequencing errors, only the *k*-mers which appear at least *d* times are kept (*solidk*-mers). We experimentally set *d* to 3. Classical methods that retrieve solid *k*-mers are based on hash tables [18], and their memory usage scale linearly with the number of distinct *k*-mers. To deal with reverse-complementation, *k*-mers are identified to their reverse-complements.

### *k*-mer counting

To avoid using more memory than the whole structure, we implemented a novel, constant-memory *k*-mer counting procedure. The multi-set of all *k*-mers present in the reads is partitioned and partitions are saved to disk. Then, each partition is separately loaded in memory in a temporary hash table. The *k*-mer counts are returned by traversing each hash table. Low-abundance *k*-mers are filtered. This approach permits to count all *k*-mers of a human genome dataset using only a fixed amount of memory and disk space. The algorithm is explicitly described and evaluated in another article [19].

### Graph traversal

We implemented in Minia a graph traversal algorithm that constructs a set of contigs (gap-less sequences). The Bloom filter and the *cFP* structure are used to determine neighbors of each node. The marking structure records already traversed nodes. A bounded-depth, bounded-breadth BFS algorithm (following Property 2 in [16]) is performed to traverse short, locally complex regions. Specifically, the traversal ignores tips (dead-end paths) shorter than 2*k*+1 nodes. It chooses a single path (consistently but arbitrarily), among all possible paths that traverse graph regions of breadth ≤20, provided these regions end with a single node of depth ≤500. These regions are assumed to be sequencing errors, short variants or short repetitions of length ≤500 bp. The breadth limit prevents combinatorial blowup. Note that paired-end reads information is not taken into account in this traversal. In a typical assembly pipeline (e.g. [8]), a separate program (*scaffolder*) can be used to link contigs using pairing information. Also, a gap-filling step (e.g. GapCloser in SOAPdenovo [7]) is typically used to fill the gaps between contigs in scaffolds.

## Results and discussion

Throughout the Results section, we will refer to the N50 metric of an assembly (resp. NG50) as the longest contig size, such that half the assembly (resp. half the reference) is contained in contigs longer than this size.

### On the usefulness of removing critical false positives

To test whether the combination of the Bloom filter and the *cFP* structure offers an advantage over a plain probabilistic de Bruijn graph, we compared both structures in terms of memory usage and assembly consistency. We retrieved 20 million *E. coli* short reads from the Short Read Archive (SRX000429), and discarded pairing information. Using this dataset, we constructed the probabilistic de Bruijn graph, the *cFP* structure, and marking structure, for various Bloom filter sizes (ranging from 5 to 19 bits per *k*-mer) and *k* = 23 (yielding 4.7 M solid *k*-mers).

We measured the memory usage of both structures. For each, we performed an assembly using Minia with exactly the same traversal procedure. The assemblies were compared to a reference assembly (using MUMmer), made with an exact graph. The percentage of nucleotides in contigs which aligned to the reference assembly was recorded.

**Figure 3:**
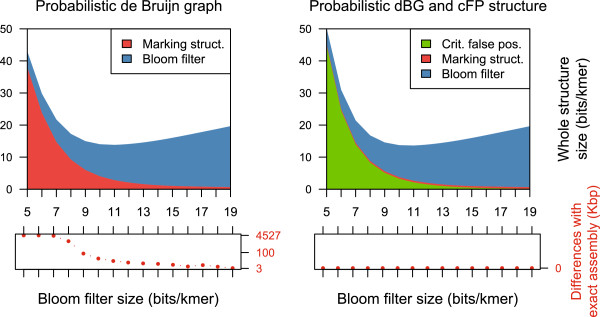
**Data structure sizes for the probabilistic de Bruijn graph.** Data structure sizes (Bloom filter, marking structure, and *cFP* if applicable) for the probabilistic de Bruijn graph with (top right) and without the *cFP* structure (top left), for an actual dataset (E. coli, *k* = 23). All plots are in function of the number of bits per *k*-mer allocated to the Bloom filter. Additionally, the difference is shown (bottom left and bottom right) between a reference assembly made using an exact de Bruijn graph, and an assembly made with each structure.

Figure 3 shows that both the probabilistic de Bruijn graph and our structure have the same optimal Bloom filter size (11 bits per *k*-mer, total structure size of 13.82 bits and 13.62 per *k*-mer respectively). In the case of the probabilistic de Bruijn graph, the marking structure is prominent. This is because the graph has a significant amount of complex *k*-mers, most of them are linked to false positive nodes. For the graph equipped with the *cFP* structure, the marking structure only records the actual complex nodes; it occupies consistently 0.49 bits per *k*-mer. Both structures have comparable memory usage.

However, Figure 3 shows that the probabilistic de Bruijn graph produces assemblies which strongly depend on the Bloom filter size. Even for large sizes, the probabilistic graph assemblies differ by more than 3 kbp to the reference assembly. We observed that the majority of these differences were due to missing regions in the probabilistic graph assemblies. This is likely caused by extra branching, which shortens the lengths of some contigs (contigs shorter than 100 bp are discarded).

Below ≈9 bits per *k*-mer, probabilistic graph assemblies significantly deteriorate. This is consistent with another article [13], which observed that when the false positive rate is over 18% (i.e., the Bloom filter occupies ≤4 bits per *k*-mer), distant nodes in the original graph become connected in the probabilistic de Bruijn graph. To sum up, assemblies produced by the probabilistic de Bruijn graph are prone to randomness, while those produced by our structure are exact.

### *de novo* assembly

#### Complete human genome

We assembled a complete human genome (NA18507, SRA:SRX016231, 142.3 Gbp of unfiltered reads of length ≈ 100 bp, representing 47x coverage) using Minia. After *k*-mer counting, 2,712,827,800 solid *k*-mers (*d* = 3) were inserted in a Bloom filter dimensioned to 11.1 bits per solid *k*-mer. The *cFP* structure contained 78,762,871 *k*-mers, which were stored as a sorted list of 64 bits integers, representing 1.86 bits per solid *k*-mer. A total of 166,649,498 complex *k*-mers (6% of the solid *k*-mers) were stored in the marking structure using 4.42 bits per solid *k*-mer (implementation uses $8\lceil \frac{k}{32}\rceil$ bytes per *k*-mer). Table 1 shows the time and memory usage required for each step in Minia.

**Table 1:** Details of steps implemented in Minia

**Step**	**Time (h)**	**Memory (Gb)**
*k*-mer counting	11.1	Constant (set to 4.0)
Enumerating positive extensions	2.8	3.6 (Bloom filter)
Constructing *cFP*	2.9	Constant (set to 4.0)
Assembly	6.4	5.7 (Bloom f.+ *cFP*
		+ mark. struct.)
Overall	23.2	5.7

Details of steps implemented in Minia, with wall-clock time and memory usage for the human genome assembly. For constant-memory steps, memory usage was automatically set to an estimation of the final memory size. In all steps, only one CPU core was used.

We compared our results with assemblies reported by the authors of ABySS [8], SOAPdenovo [7], and the prototype assembler from Conway and Bromage [9]. Table 2 shows the results for four classical assembly quality metrics, and the time and peak memory usage of the compared programs. We note that Minia has the lowest memory usage (5.7 GB), seconded by the assembler from Conway and Bromage (32 GB). The wall-clock execution time of Minia (23 h) is comparable to the other assemblers; note that it is the only single-threaded assembler. The N50 metric of our assembly (1.2 kbp) is slightly above that of the other assemblies (seconded by SOAPdenovo, 0.9 kbp). All the programs except one assembled 2.1 Gbp of sequences.

**Table 2:** de novo human genome (NA18507) assemblies

**Method**	**Minia**	**C. & B.**	**ABySS**	**SOAPdenovo**
Value of *k* chosen	27	27	27	25
Number of contigs (M)	3.49	7.69	4.35	-
Longest contig (kbp)	18.6	22.0	15.9	-
Contig N50 (bp)	1156	250	870	886
Sum (Gbp)	2.09	1.72	2.10	2.08
Nb of nodes/cores	1/1	1/8	21/168	1/16
Time (wall-clock, h)	23	50	15	33
**Memory (sum**	**5.7**	**32**	**336**	**140**
**of nodes, GB)**				

*de novo* human genome (NA18507) assemblies reported by our assembler (Minia), Conway and Bromage assembler [9], ABySS [8], and SOAPdenovo [7]. Contigs shorter than 100 bp were discarded. Assemblies were made without any pairing information.

We furthermore assessed the accuracy of our assembly by aligning the contigs produced by Minia to the GRCh37 human reference using GASSST [20]. Out of the 2,090,828,207 nucleotides assembled, 1,978,520,767 nucleotides (94.6%) were contained in contigs having a full-length alignment to the reference, with at least 98% sequence identity. For comparison, 94.2% of the contigs assembled by ABySS aligned full-length to the reference with 95% identity [8].

To evaluate another recent assembler, SparseAssembler [12], the authors assembled another dataset (NA12878), using much larger effective *k* values. SparseAssembler stores an approximation of the de Bruijn graph, which can be compared to a classical graph for *k*^′^=*k*+*g*, where *g* is the sparseness factor. The reported assembly of the NA12878 individual by SparseAssembler (*k*+*g* = 56) has a N50 value of 2.1 kbp and was assembled using 26 GB of memory, in a day. As an attempt to perform a fair comparison, we increased the value of *k* from 27 to 51 for the assembly done in Table 2 (*k* = 56 showed worse contiguity). The N50 obtained by Minia (2.0 kbp) was computed with respect to the size of SparseAssembler assembly. Minia assembled this dataset using 6.1 GB of memory in 27 h, a 4.2× memory improvement compared to SparseAssembler.

#### Chromosome 14 of the human genome

In order to evaluate the quality of the results produced by Minia more accurately, we assembled the human chromosome 14 (88 Mbp ungapped) separately. The Illumina dataset is from the GAGE benchmark [21] (100 bp reads, all short paired-end libraries). Pairing information was not used in Minia. To establish a fair comparison with Minia, we selected two assemblies from GAGE (made with ABySS and Velvet, downloaded from the GAGE website) for which contigs were constructed without using pairing information. All the assemblies were done with a *k*-mer size of 31, as chosen by the authors of GAGE. Additionnaly, we executed Minia with *k* = 47, as this *k*-mer size was experimentally found to provide better results than *k* = 31.

QUAST v1.3 was executed with the --gage option to evaluate the quality of the contigs of each assembly. Table 3 shows several contiguity, coverage and quality metrics computed using the same reference genome and QUAST command-line for the three assemblies. The large misassemblies row shows the number of positions in the assembled contigs for which the left and right flanking sequences align either over 1 kbp away from each other, or on different strands or chromosomes. The local misassemblies row shows the number of positions in the assembled contigs for which the left and right flanking sequences are distant from each other by 90 bp to 1 kbp.

**Table 3:** ***de novo *human genome chromosome 14 assemblies**

**Assembly**	**Minia**		**ABySS**	**Velvet**
Value of *k* chosen	47	31	31	31
Number of contigs (k)	48.5	56.1	51.9	45.6
Longest contig (kbp)	28.9	26.5	30.0	27.9
Contig NG50 (kbp)	2.8	1.7	2.0	2.3
Large misassemblies	11	3	20	385
Local misassemblies	25	27	158	867
Coverage (%)	92.4	81.9	82.2	84.4
Unaligned contigs length (kbp)	33.1	13.5	23.6	564.5

*de novo* human genome chromosome 14 assemblies reported by our assembler (Minia), compared to Velvet and ABySS assemblies from the GAGE benchmark. QUAST was used to evaluate all three assemblers.

On this dataset, Minia with *k* = 31 produced an assembly of lower contiguity and better accuracy to ABySS and Velvet. Minia with *k* = 47 produced a better assembler over all metrics than the other assemblers run with *k* = 31. Overall, the quality of assemblies produced with Minia can be considered satisfactory with respect to contigs made from state of the art assemblers. It should be noted that in the GAGE benchmark, other methods (e.g. SOAPdenovo, Allpaths, etc..) produced assemblies of much higher contiguity. These methods performed post-processing steps (scaffolding and gap-filling), usually after completing an initial contigs construction phase with a classical de Bruijn graph.

## Conclusions

This article introduces a new, space-efficient representation of the de Bruijn graph. The graph is implicitly encoded as a Bloom filter. A subset of false positives, those which introduce false branching from true positive nodes, are recorded in a separate structure. A new marking structure is introduced, in order for any traversal algorithm to mark which nodes have already been visited. The marking structure is also space-efficient, as it only stores information for a subset of *k*-mers. Combining the Bloom filter, the critical false positives structure and the marking structure, we implemented a new memory-efficient method for *de novo* assembly (Minia).

To the best of our knowledge, Minia is the first method that can create contigs for a complete human genome on a desktop computer. Our method improves the memory usage of de Bruijn graphs by two orders of magnitude compared to ABySS and SOAPdenovo, and by roughly one order of magnitude compared to succinct and sparse de Bruijn graph constructions. Furthermore, the current implementation completes the assembly in 1 day using a single CPU thread.

De Bruijn graphs have more NGS applications than just *de novo* assembly. We plan to port our structure to replace the more expensive graph representations in two pipelines for reference-free alternative splicing detection, and SNP detection [4,6]. We wish to highlight three directions for improvement. First, some steps of Minia could be implemented in parallel, e.g. graph traversal. Second, a more succinct structure can be used to mark complex *k*-mers. Two candidates are Bloomier filters [22] and minimal perfect hashing.

Third, the set of critical false positives could be reduced, by exploiting the nature of the traversal algorithm used in Minia. The traversal ignores short tips, and in general, graph regions that are eventually unconnected. One could then define *n*-th order critical false positives (*n*-*cFP*) as follows. An extension of a true positive graph node is a *n*-*cFP* if and only if a breadth-first search from the true positive node, in the direction of the extension, has at least one node of depth *n*+1. In other words, false positive neighbors of the original graph which are part of tips, and generally local dead-end graph structures, will not be flagged as critical false positives. This is an extension of the method presented in this article which, in this notation, only detects 0-th order critical false positives.

Simultaneously and independently of the present work, Bowe *et al.*[23] have proposed a de Bruijn graph representation using 4+*o*(1) bits per edge. Their data structure is more succinct than ours, and at the same time more complex and difficult to implement. Since their structure has not been fully implemented yet, it is not possible to compare the practical performance with Minia. However, this new advance is very interesting and raises the question of whether their structure is the most succinct way to represent a de Bruijn graph.

## Endnote

^a^ Source code available at http://minia.genouest.org/.

## Competing interests

Both authors declare that they have no competing interests.

## Authors’ contributions

RC and GR equally contributed to the data structure design, software and manuscript. Both authors read and approved the final manuscript.
